# Correcting misinformation about the Russia-Ukraine War reduces false beliefs but does not change views about the War

**DOI:** 10.1371/journal.pone.0307090

**Published:** 2024-09-23

**Authors:** Ethan Porter, R. Bailey Scott, Thomas J. Wood, Raushan Zhandayeva

**Affiliations:** 1 Institute for Data, Democracy and Politics, George Washington University, Washington, D.C., United States of America; 2 Department of Political Science, Ohio State University, Columbus, OH, United States of America; Thammasat University Institute of East Asian Studies, THAILAND

## Abstract

We report results from simultaneous experiments conducted in late 2022 in Belarus, Estonia, Kazakhstan, Russia and Ukraine. The experiments focus on fact-checking misinformation supportive of Russia in the Russia-Ukraine War. Meta-analysis makes clear that fact-checking misinformation reduces belief in pro-Kremlin false claims. Effects of fact-checks are not uniform across countries; our meta-analytic estimate is reliant on belief accuracy increases observed in Russia and Ukraine. While fact-checks improve belief accuracy, they do not change respondents’ attitudes about which side to support in the War. War does not render individuals hopelessly vulnerable to misinformation—but fact-checking misinformation is unlikely to change their views toward the conflict.

## Introduction

The Russia-Ukraine War has been marked by misinformation since the start, when false claims about the rise of Nazis and persecution of Russian minorities in Ukraine were offered as justification for Russia’s invasion [1, 2]. As the conflict has gone on, misinformation has continued to circulate, with false claims about, among other topics, NATO-sponsored bio-weapons labs in Ukraine, mass persecution and genocide of ethnic Russians, and Ukrainian state “lies” about Russian atrocities [3]. When news stories broke about civilian targeted killings and atrocities in Bucha, the Russian media claimed that Ukraine was orchestrating fake images and videos about the massacre [4]. Later, as Russian casualties piled up, the misinformation machine touted Russian victories, claiming that Ukrainian casualties were far greater [5]. This type of blatant misinformation promotes messages that sew discontent, bolster the Russian position in the war, and paint Ukraine and the West as the aggressors in the conflict [3, 6].

What, if anything, can be done in response to pro-Kremlin misinformation? Scholars have identified a variety of effective tools for responding to misinformation in general [7–10]. We study how one such tool—fact-checking—fares during wartime, targeting misinformation favoring one of the belligerent states, with participants recruited from those states and their neighbors. (Note that, in this paper, we use the term “misinformation” encompass both clear state-sponsored falsehoods and falsehoods for which intention and provenance are unknown. We use the term “pro-Kremlin” to be clear we are discussing misinformation flattering a particular regime).

Specifically, we report results from five pre-registered experiments, administered simultaneously in Belarus, Estonia, Kazakhstan, Russia and Ukraine, which measure the effects of fact-checks on belief accuracy (i.e., the extent to which people believe misinformation) and downstream political beliefs (i.e., people’s attitudes about which side to support in the War). In short, we evaluate whether fact-checks which target pro-Kremlin misinformation can change people’s belief in false claims about the War, and if doing so changes in their broader views about the War.

The tested fact-checks were Russian-language fact-checks produced by leading area fact-checking organizations. Table 1 summarizes the fact-checks and the targeted misinformation. The S1 Appendix contains more detailed summaries. The fact-checks were not edited or modified in any way. By relying on real-world fact-checks, we are able to evaluate what is actually being done in response to misinformation. In many cases, the misinformation underlying the fact-checks was quite popular. In S1 and S2 Tables in S1 Appendix, we present available evidence on the popularity and prevalence of our tested misinformation and fact-checks, respectively.

**Table 1:** Summaries of misinformation topics and fact-checking sources, by country.

Misinformation claim	Fact check source
*Belarus trials*	
Ukraine did not formally register its borders with the UN in 1991	factcheck.kz
Footage of alleged Russian war crimes in Bucha is fake	Deutsche Welle
Textbooks found in a Ukrainian school promote Nazism	Re:Baltica
*Estonia trials*	
Estonia is protecting Darya Dugina’s murderer	PropaStop
Estonian fascists plan to forcibly integrate Russians	PropaStop
Baltic countries sabotaged the Nord Stream pipeline	PropaStop
*Kazakhstan trials*	
Ukrainian refugees have created a teacher shortage in Germany	factcheck.kz
Luhansk and Donetsk Republics have an official representative office in Finland	factcheck.kz
Footage of a documentary was presented as Russian atrocities in Ukraine	factcheck.kz
*Russia trials*	
Ukraine trades in grain in exchange for weapons	Re:Baltica
The Guardian denied Russian involvement in Bucha killings	Re:Baltica
Russia has never started or been the aggressor in a war	Re:Baltica
*Ukraine trials*	
Donbas inhabitants subjected to genocide by Ukraine	factcheck.kg
Ukraine is raising money for an atomic bomb	factcheck.kg
The Pentagon stated there are 46 US-funded biolabs in Ukraine	Re:Baltica

While fact-checking may be popularly associated with Western media, it is an increasingly global journalistic practice [11]. Fact-checks are distinct from succinct “debunks,” as they present readers with more evidence in the service of rebutting false claims (although the effects of debunks and fact-checks are substantively similar; see [12]). Although some earlier research suggested factual corrections have null effects or can even backfire, entrenching false beliefs, [13], more recent findings show that exposure to corrections reliably improves belief accuracy [14–17] and do not backfire [18]. Yet with few exceptions [19, 20], these findings have depended on samples from a limited set of peaceful, prosperous “WEIRD” states [21].

Especially in Russia, exposure to fact-checks of pro-Kremlin misinformation would seem to be capable of provoking “worldview backfire,” wherein subjects respond to corrective information that targets their most cherished beliefs by becoming less accurate [18]. Recent scholarly evidence, however, has coalesced around the idea that direct factual responses to misinformation, often delivered under the guise of fact-checks, can lead individuals to reject misinformation and evince more accurate beliefs [22]. Success at mitigating false beliefs generated by misinformation has been shown across a wide swath of the globe [23], including in countries in the Global South [20, 24]. Yet fact-checking pro-Kremlin misinformation in Russia, during wartime, stands as a hard test of the global efficacy of corrections. In our pre-registration document (available in the S1 Appendix), we specified that we expected exposure to factual corrections would improve belief accuracy, reducing belief in the corrected misinformation (H1).

We viewed these experiments as an opportune time to investigate whether increasing the quantity of corrective evidence would change participants’ attitudes toward the War. While prior work has shown that exposure to *one* fact-check has nonexistent to microscopic effects on attitudes [25], other studies find that exposure to multiple fact-checks at once can indeed shift attitudes, albeit not uniformly [26]. Providing multiple fact-checks simultaneously amounts to providing a larger exogenous shock to those participants’ media diet than just one fact-check.

We therefore randomized not only whether participants would see a fact-check, but how many they would see over the course of our study (between 0 and 3). In doing so, we would be better-positioned to measure the effects of the quantity of fact-checks seen on attitudinal changes to the War than we would with a conventional one-shot fact-checking experiment. We pre-registered a research question focused on the potential for multiple fact-checks to move attitudes (RQ1a-RQ1b). In the pre-registration document, we also made clear we would study how effects on belief accuracy and support for the War would differ across countries (RQ2).

Overall, when accounting for respondents across all five experiments (n = 3,946), we find that exposure to corrections reduces belief in pro-Kremlin misinformation by .21 points on a 5-point scale (p < .05). Intriguingly, this finding is strongly reliant on our participants in Russia and Ukraine. Participants from *both* countries did not backfire, instead moving in the direction of greater accuracy. However, we do not find evidence that exposure to fact-checks changes views about the War. Instead, correcting misinformation reduces belief in pro-Kremlin misinformation, without having concomitant effects on broader attitudes about the War or its participants. On the one hand, this extends prior findings about the power of fact-checks to improve belief accuracy without meaningfully changing downstream attitudes [16, 25]. On the other hand, it throws cold water on popular assumptions about the power of misinformation during war, and what can reasonably be expected from responding to it.

## Experimental design and outcome measures

The experiments launched simultaneously in Belarus, Estonia, Kazakhstan, Russia, and Ukraine in November 2022. In each country, respondents were exposed to between 0–3 factual corrections of pro-invasion misinformation in Russian. The fact-checks were produced by Russian-language fact-checking organizations operating in each country. To maximize the realism of the treatments [27], participants were presented with the fact-checks as they originally appeared in Russian, without modification. We offer more details in the S1 Appendix and in Table 1.

In each country, the experiments proceeded identically. First, participants answered general demographic questions, political ideology and psychology questions, as well as an attention check question, with the latter modeled on [28]. Participants who failed the attention check question did not proceed to treatment.

**Fig 1:**
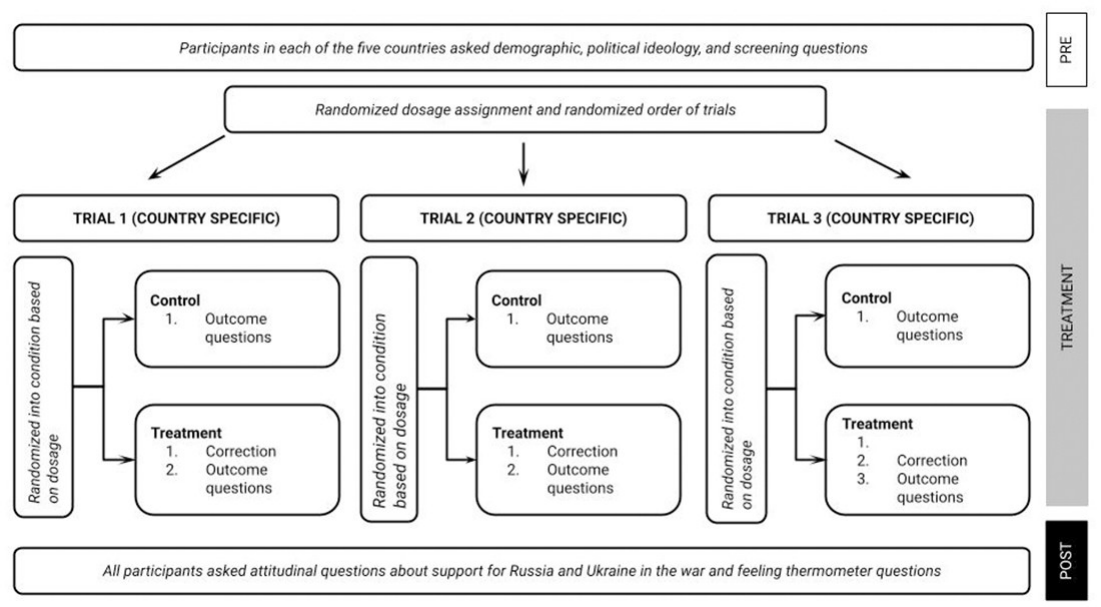
Summary of experimental design across five different countries with country-specific trials.

Then, in the treatment phase, participants were enrolled in three trials. In each trial, participants saw a fact-check and answered an outcome question meant to gauge their belief in the misinformation being fact-checked, or just answered the outcome question. Participants were randomized to see 0–3 fact-checks over the course of the study. To be clear, the number of fact-checks was randomly assigned, as was the specific fact-checks seen, as well as the order of the trials themselves. After the end of all three trials, participants answered outcome questions about their views about the Russia-Ukraine war. Fig 1 depicts the design.

The fact-checks used in each country were unique. While each of the corrections came from a different source, all corrections specifically focused on popular Russian misinformation rhetoric. The fact-checks focused on misinformation that vilifies Ukraine, portrays Ukraine as stooges of the West, exaggerates justifications for Russia’s invasion, and sews discontent about the Ukrainian war. At times, the corrections are responsive to locally-targeted misinformation; other times, the misinformation is more sweeping. Moreover, while most of the topics in some way related back to the Russo-Ukrainian war, some topics had more specific relation to the target country itself. For example, in Estonia, one fact-check and its outcome question focused on the murder of Daria Dugina. Dugina was the daughter of Aleksandr Dugin, an ideological godfather to the “Eurasinist” movement that informs many of Putin’s geopoltical postures [29]; the Russian media machine has spun a vast web of conspiracies about her murder.

Table 1 summarizes the stimuli used in each country; more detailed summaries appear in the S1 Appendix. The S1 Appendix also describes how tested fact-checks relate to the long legacies of Russian misinformation. In selecting fact-checks to test, we sought to include a diverse mix of credible, Russian-language fact-checking organizations that had targeted pro-Russian misinformation. Overall, all tested fact-checks targeted misinformation by Russia and Russia-aligned media and proxy organizations meant to bolster Russian political and military aims.

To measure belief accuracy, and consistent with prior work in this literature, participants were asked to share their level of agreement with a statement summarizing the misinformation. Specifically, respondents were asked: “To what extent do you agree or disagree with the following statement?” The exact statements can be found in Table 1; available responses ranged from “Strongly agree” to “Strongly disagree,” on a 1–5 scale.

To measure support for the war, we asked respondents their level of support for each side in the war (i.e., Russia and Ukraine), on separate 1–5 agree-disagree sliders, ranging “Strongly Disagree” to “Strongly Agree.” Respondents could drag the slider along the 1–5 scale; there was not separate text about each choice. We also presented respondents with standard 0–100 feeling thermometers for eight targets; as pre-registered, we only inspected evaluations to Vladimir Putin, Volodymyr Zelensky, Russia, and Ukraine, with the remaining four serving as decoy items.

This research was approved by the George Washington University Institutional Review Board, IRB #NCR224480. Participants provided electronic informed consent before participating. The surveys began on November 17 2022 and concluded on December 2 2022.

## Results

Participants were recruited via Lucid, a well-known provider of online survey respondents that a) generates results comparable to those obtained on other platforms and b) can approximate population demographic benchmarks [30]. To mitigate concerns about bots and/or otherwise inattentive respondents, such as those voiced by [31], prior to treatment we administered an attention check modeled on that used by [28]; those who failed this check were not permitted to proceed. S1 Fig in S1 Appendix reports the time spent on the survey, by corrections seen and respondent’s country. In each country, subjects spent more time on the survey when they saw more corrections, consistent with the average subject being proportionally attentive to material they saw in the survey.

Further evidence of data quality can be found in the S1 Appendix, where we compare the geographic distribution of our survey respondents, as well as their ages and genders, to available population-level details about the same. Especially on the gender and geographic dimensions, respondents in all five states were distributed in ways broadly comparable to the populations of which they were a part. Ultimately, while these differences do not affect treatment effects, compared to the populations, our samples are younger, have slightly skewed gender distributions and naturally only include Internet users.

S3 Table in S1 Appendix presents the number of survey respondents by the number of corrections to which they were assigned to see in each country; this offers evidence of successful randomization. To be clear, the data are not infallible; in Belarus, we observed about 10% of participants declining to answer questions. However, this did not occur in the other four countries.

**Fig 2:**
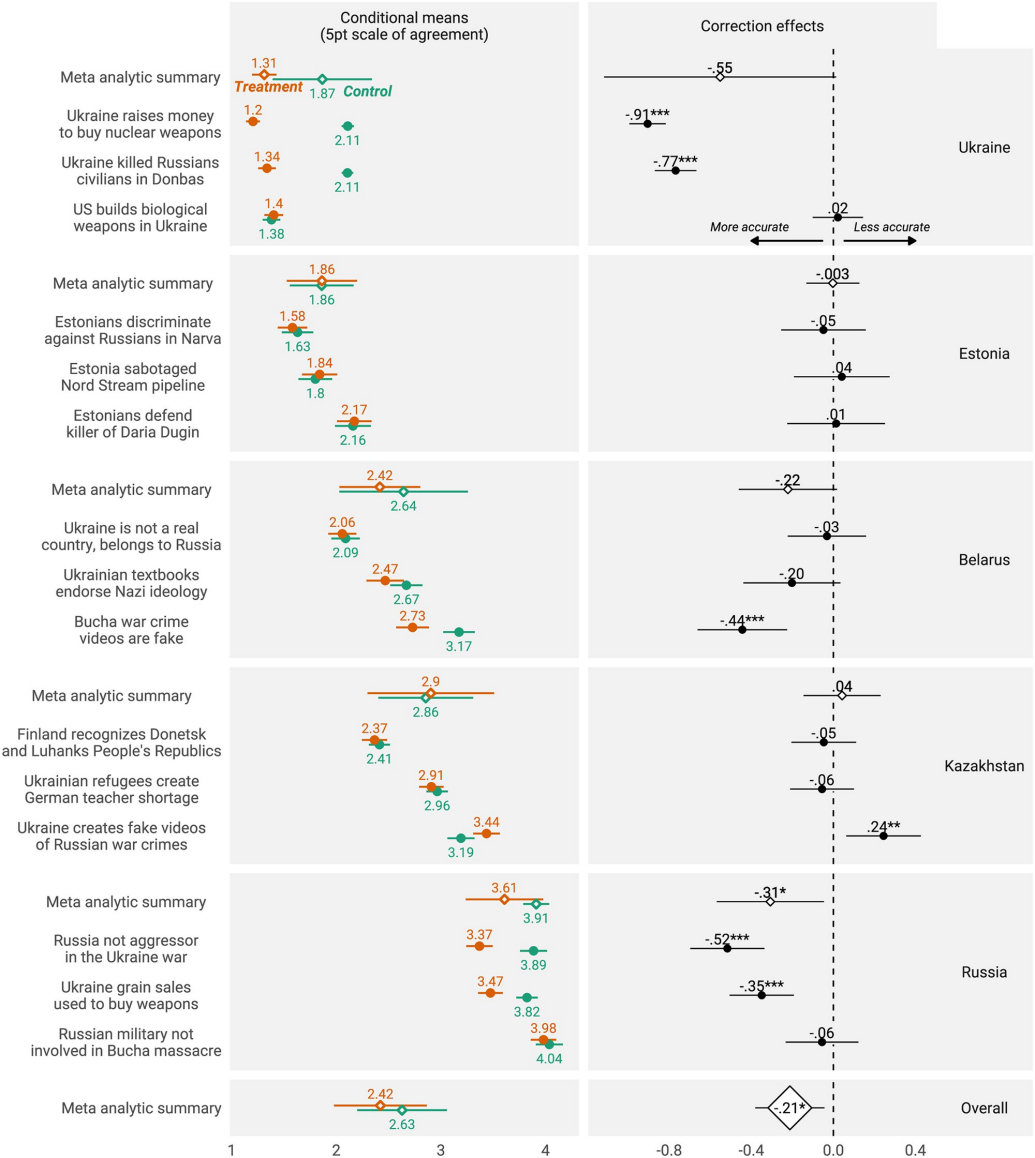
Correction effects. The left column reports conditional mean agreement, by issues and countries. Hollow points report meta-analytic summaries. The right column reports correction effect (with agreement in treatment subtracted from agreement among control participants), and their 95% confidence intervals. Negative correction estimates report improved accuracy-positive values decreased accuracy. *Note*: * p <.05, ** p <.01, *** p <.001.

Consistent with our pre-registered hypothesis (H1) and analytic strategy, we observe exposure to factual corrections improving belief accuracy. Across all five countries and experiments, the combined meta-analytic estimate is .21 (p < .05) on our five point scale, in the direction of greater accuracy (S11 Table in the S1 Appendix). To be clear, this estimate accounts for all fifteen tested fact-checks in all five countries, treating each trial as a discrete experiment. Fig 2 displays results. Meta-analytic estimates appear in the bottom row; country-by-country results appear above. We always use two-tailed tests.

As Fig 2 illustrates, belief accuracy was not observed uniformly across all five countries. We were able to detect belief accuracy increases in Russia, among participants close to the conflict. This echoes [32], who theorized that as proximity to conflict increases, so do does capacity to separate truth from fiction. Further evidence in favor of this hypothesis comes from our Ukraine results; the p-value for the meta-analytic estimate in Ukraine is .0557, vanishingly close to our pre-registered threshold. Some of the inter-country differences may be due to differing sample sizes, which in turn impact our ability to detect effects or not; some are also likely due to underlying differences between the countries. Note that, for one fact-check in Kazakhstan, we observe evidence of backfire, whereby exposure to the fact-check reduces belief accuracy. On the whole, however, and contrary to speculation, it is *not* the case that war precludes those who experience it most intimately from distinguishing fact and fiction.

**Fig 3:**
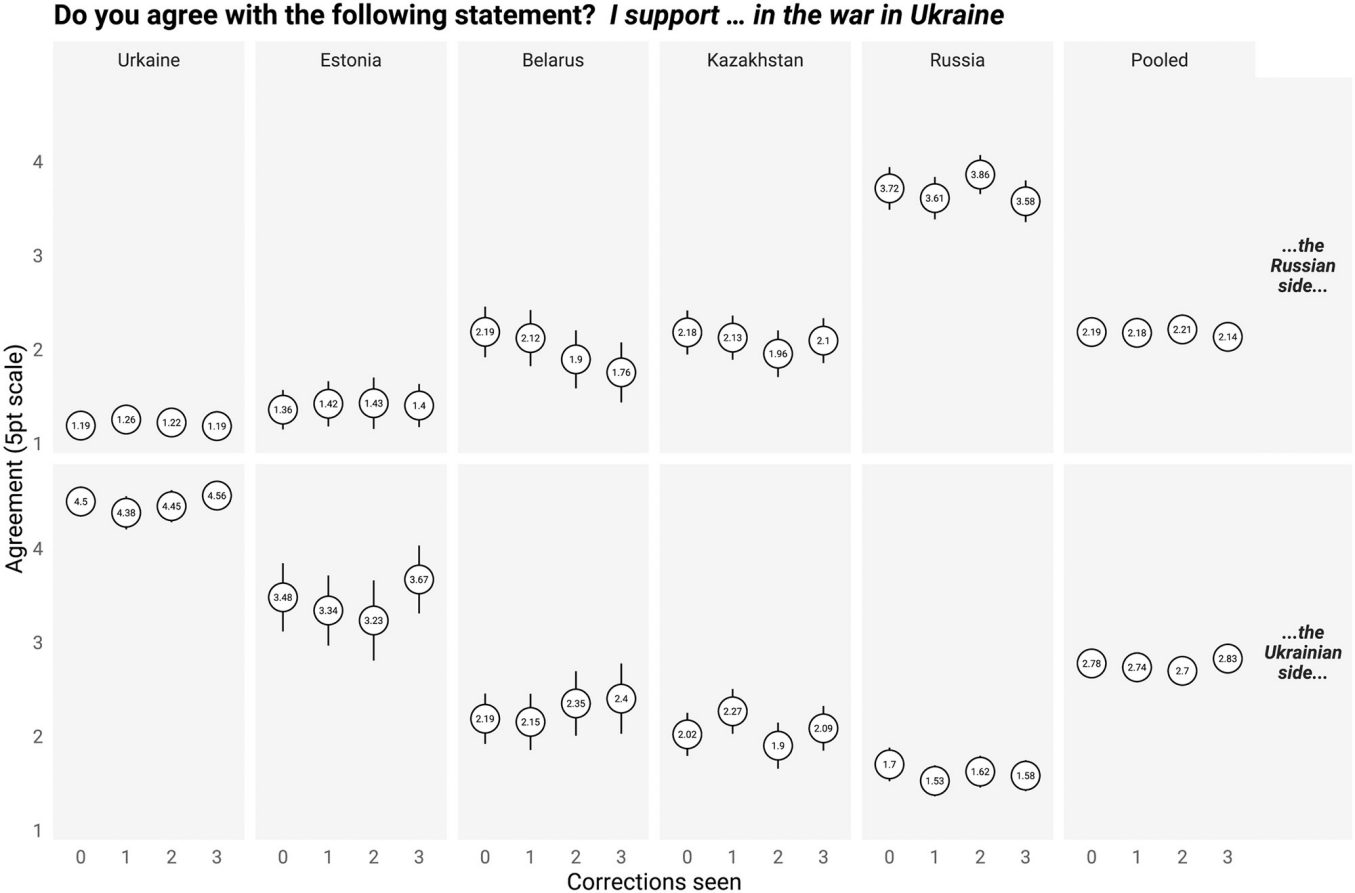
Corrections and attitudes toward the war.

What about effects on attitudes? Can higher quantities of fact-checks cause individuals to change their views about the War? It does not appear so. On all our attitudinal outcomes, exposure to increasing quantities of corrections did not measurably change attitudes. Fig 3 displays the effects of the number of corrections seen and support for the War; S3 Fig in S1 Appendix displays effects on the specific feeling thermometers of interest. Complete regression results for all attitudinal outcomes can be found in the S5-S18 Tables in S1 Appendix. Support for the Russian side is higher in Russia and lowest in Ukraine, and vice versa, while other outcomes follow the same pattern. This corroborates other work [33, 34].

Exposure to corrections does not affect attitudes toward the war, including as the number of corrections increase (S19 and S20 Tables in S1 Appendix). As an exploratory measure (not pre-registered), we also examined whether the net difference between support for Ukraine and support for Russia changed as a result of correction exposure; in any country and in aggregate, it did not. Becoming more accurate amidst war, and rejecting misinformation, does not coincide with changes to one’s views about the War.

**Fig 4:**
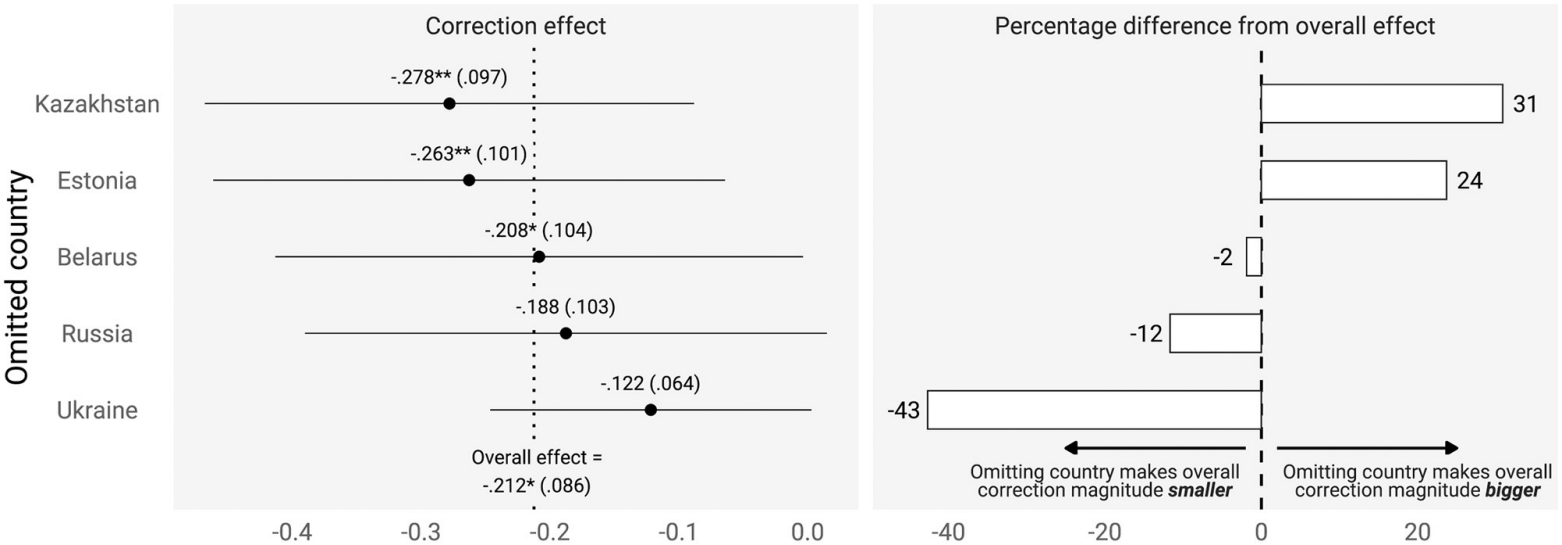
Effect of omitting countries on the overall correction effect. The left panel shows, for each country (indicated by the row labels), what overall meta-analytic correction effect is estimated when omitting that country. The dashed vertical line in the left panel reports the overall correction effect when no country is omitted. The right panel depicts the percentage change in the *magnitude* of the overall correction effect, when the row country is omitted.

To what extent are our results affected by the mixture of countries in our study? To find out, we re-estimated our main correction effect meta-analytic model five times, each time omitting a different country. Fig 4 displays the result of each re-estimation and shows the percentage difference between the overall correction effect, with all five countries accounted for, and each re-estimated effect, with only four. Had we omitted Kazakhstan or Estonia, the observed effect would have strengthened; omitting Belarus would have had virtually no impact; omitting Russia or Ukraine would have weakened the effect. Indeed, had we not included Russia, the observed effect would be 12% smaller, while not including Ukraine would have shrank the effect by 43%. Our conclusion that correcting pro-Kremlin misinformation improves belief accuracy is strongly reliant on the Ukrainian and Russian samples.

One possible concern with our results is that they are affected by selection bias, with the suspicion being that people in these countries who elect to participate in online surveys must be meaningfully distinct from those who do not. While we make no claims to representativeness, several features of our sample and data should temper such concerns. First, as previously mentioned, in the S4-S10 Tables in S1 Appendix, we compare age, gender and geographic region data in our sample to available data for each country studied. Across countries, we observe that the samples are broadly similar to population data on these dimensions (though less so on gender). Furthermore, other studies of factual corrections have found no difference between estimates obtained on convenience samples and those obtained on nationally representative samples [16]. Second, the control data from the attitudes outcomes makes clear that respondents have attitudes akin to what one would reasonably expect and that mirror other available data. If, say, our Ukrainian sample included a large number of respondents openly critical of the Ukrainian side, then the control attitude data from the Ukraine sample would be more skeptical of the Ukraine side than it is. The inverse is true for the Russian sample. To be clear, we cannot eliminate the possibility that our data would be different had we obtained a representative sample. But the available evidence suggests that our results are not wholly dependent on unusual features of our samples.

## Discussion

Five experiments, conducted simultaneously in five countries in and in close proximity to the Russia-Ukraine War, show that, in one respect, concerns about the effects of misinformation during wartime are unwarranted. It has long been argued that misinformation disseminated during war may prevent people from discerning truth from falsehood [35]. One observer of the current conflict posits that pro-Kremlin misinformation efforts are motivated by the Kremlin’s interest in leaving “people simply confused about what’s true and what’s not” [36]. If this is the motivation, they do not succeed. In areas bombarded with pro-Kremlin misinformation—including in Russia itself—people are quite capable of being made more accurate by corrective evidence. People typically respond to fact-checks of that misinformation by becoming more accurate. Even during wartime, worldview backfire does not rear its head.

At the same time, fact-checks do not affect certain attitudes about the War. This mixed conclusion—that corrections improve belief accuracy while leaving attitudinal outcomes nearly entirely unaffected—has been observed repeatedly on Western samples [25]. The present findings extend this conclusion to a surprising new domain, that of people close to active conflict. Our work thus contributes to a growing body of literature that finds factual corrections have miniscule-to-nonexistent effects on attitudes (as summarized in [37]). Of course, it is possible that other attitudes, not studied here, are affected by fact-checks. But the attitudinal outcomes we measure do not move. 

Furthermore, as we demonstrate in this paper, even increasing the quantity of corrections does not alter this conclusion. Insofar as reducing belief in misinformation is a worthwhile objective in and of itself, then correcting misinformation is worth doing during wartime. But if the objective is to shift related attitudes, then correcting misinformation, even repeatedly, may not be the most advisable tactic.

We must emphasize that our results are contingent: *If* individuals living in conflict see corrections of widely-circulating misinformation, they will, on average, be moved to hold more accurate beliefs. While governments and other institutions have already devoted considerable efforts to responding to Russian misinformation, we are skeptical that many people have seen actually fact-checks of misinformation. In the U.S., web-browsing data shows that the disconnect between those who consume misinformation and those who consume fact-checks is extraordinarily large. Only 2.7% of individuals who consume a particular piece of misinformation go on to consume the corresponding factual correction [38]. We suspect similar patterns may play out elsewhere, including in the countries studied here. Large-scale efforts to correct misinformation may improve belief accuracy, but they are hardly assured of getting in front of the people who may need such efforts most. More research identifying means of increasing readership of fact-checks [39], as well as measuring the determinants of exposure to corrective information [40], is urgently needed.

We cannot rule out the possibility that participants—especially in Russia—felt that revealing their true attitudes about the War would be precarious in a way that merely updating their factual beliefs would not be. Media reports have relayed stories of ordinary Russians reporting each other for perceived disloyalty to the government and the War [41]. In our study, even if fact-checks yielded the desired effect, respondents may have felt pressure not to disclose them (consistent with [42–44]). Future research should make careful use tools designed to account for this possibility, such as list experiments (as discussed in [45]).

There are several further limitations of the present study worth remarking upon. One has to do with statistical power; we studied the maximum number of participants our survey vendor was able to recruit, which, under certain assumptions about effect size, may have left us comparatively less well-powered in certain countries. In S2 Fig in S1 Appendix, we present country-by-country results on the relationship between alpha level, sample size and effect size on statistical power. For all but the smallest effects, we were well-powered in all four countries. For the second smallest effects, we were less well-powered in Estonia but reasonably well-powered elsewhere. By the standards of [12], we were well-powered in every country. Still, greater statistical power would have facilitated more precision and better-facilitated comparisons between countries. Another has to do with language. Across countries, our experiments proceeded in Russian; those who did not speak Russian were not eligible to participate. This certainly affected recruitment and may have affected effects. That said, in other fact-checking work which has evaluated the same misinformation and fact-checks across multiple languages [19], the results across languages have been consistent.

Still another limitation has to do with the set of fact-checks we included. While we designed our study to include a diverse set of Russian-language fact-checking organizations targeting prevalent pro-Russian misinformation, it is possible that the differing levels of popularity of the fact-checking sites (as exhibited in S1 Table in S1 Appendix) may have impacted our results. So too might have the reputations of the fact-checking organizations. For example, Re:Baltica may have been regarded as anti-Russian disinformation by pro-regime Russian participants; if so, the effects we do observe in Russia may be conservatived by the fact-checking source.

More broadly, our study design is captive to the misinformation and fact-checking environment of the time at which the study was conducted. Not all fact-checks, and not all misinformation, are created alike. For example, in Belarus, we inspected fact-checks of a false claim asserting that “Ukraine is not a real state.” This might be thought of as part and parcel of a false narrative, rather than false information. Future research should disentangle whether the effects of fact-checking differ depending on the role that narrative plays in the misinformation being fact-checked. It seems possible that misinformation disseminated as part of broader narratives may be more difficult to dislodge than more discrete information (e.g., false numbers about casualties), but we can only speculate at present.

While we cannot say for sure why our effects varied across treatments and countries, we do think it is worth raising several possible explanations. One has to do with the varying salience of false claims across countries; misinformation about bio-weapons in Ukraine may be received differently there than, say, misinformation about the Nord Stream pipeline in Estonia. (Consult S2 Table in S1 Appendix for details about the prevalence of our tested misinformation items). More broadly, the differences may be owed to broader cross-national differences in media systems, as studied by [46]. Another explanation concerns the varying role of government in each country. The Russian government, for example, is more likely to both suppress and spread certain instances of misinformation than the Estonian government. We cannot observe the “pre-treatment effects”, as studied by [47], that may be generated by governments and may be affecting our results. Still another explanation has to do with latent quality measures of both misinformation and fact-checks. That is, there may be subtle features of both the false claims and the fact-checks that lead them to be more (and less) persuasive. None of these explanations are mutually exclusive; more experimental research, in which these possible sources of heterogeneity are carefully randomized, is needed.

Organizations and governments that hope responding to misinformation with accurate information will, in turn, shape views toward the Russo-Ukraine War are likely to be disappointed with our findings. If anything, our evidence from Ukraine indicates that corrective information is likely to find its most receptive audience among those already predisposed to hold the producer of misinformation, in this case Russia, in poor esteem. While respondents in Russia may have been strategically adjusting their responses out of fear, the available evidence indicates that showcasing the lies of their regime has no affect on their views toward the War. Of course, this is not to say that misinformation should not be responded to; in a counterfactual environment, in which no attempt whatsoever had been made to respond to misinformation already-circulating, our observed estimates might look very different. But we cannot gather evidence from that environment. Instead, the evidence we do have shows that correcting Russian misinformation improves belief accuracy among those who live in and adjacent to the War, but has not detectable effects on attitudes toward the War itself.

## Supporting information

S1 Appendix (PDF)
