# Supplementary material for: Correcting misinformation about the Russia-Ukraine War reduces false beliefs but does not change views about the War
Source: PLoS One. 2024 Sep 23;19(9):e0307090. doi: 10.1371/journal.pone.0307090 (PMC11419341; doi:10.1371/journal.pone.0307090)
Supplement: S1 Appendix — (PDF) [file pone.0307090.s001.pdf]

Appendix

Contents

1 Detailed Summaries of Stimuli **3**

2 Tables **11**

3 Pre-registration **26**

List of Figures

S1 Time spent by participants inside the survey environment, by country and corrections seen. Labeled points report the mean duration spent on the survey. 18

S2 The statistical power for three levels of significance (indicated in column facets) and four levels of effect size (indicated by the row facets). The statistical power observed in each country is indicated by the labeled points for every combination of significance level and effect size. 19

S3 Feeling thermometer evaluations by country. Labels indicate mean evaluations. 20

List of Tables

S1 Popularity of the Fact-Checking Websites 11

S2 Prevalence of Misinformation 12

S3 The number of survey participants by country and number of corrections assigned. 13
S4 Summary Age Demographic in Survey Compared with Population Estimates . 13
S5 Summary Gender Demographic in Survey Compared with Population Estimates 13
S6 Summary comparing survey regional demographics with real Belarusian regional demographics 14
S7 Summary comparing survey regional demographics with real Estonian regional demographics 14
S8 Summary comparing survey regional demographics with real Kazakh regional demographics 15
S9 Survey regional demographics vs. Russian regional demographics (data from 2021 Russian census. See: https://rosstat.gov.ru) 16
S10 Survey regional demographics vs. Ukrainian regional demographics 17
S11 Regression estimates for correction effects. Treatment effects report the difference in factual accuracy of seeing a factual correction. The dependant quality is measured on a 5-pt scale, with higher values indicating less accurate beliefs. Intercepts are omitted from this table for simplicity, These models underpin the correction estimates in Figure 3. 21
S12 Meta analytic summaries of agreement with misinformation among subjects in control condition 22
S13 Meta analytic summaries of agreement with misinformation among subjects in correction condition 22
S14 Meta analytic summaries of difference in agreement between conditions, overall, and by country 22

S15 Effect of corrections seen on feeling thermometer evaluations of Vladimir Putin. Reference category for corrections seen variable is no corrections seen. 23
S16 Effect of corrections seen on feeling thermometer evaluations of Volodymyr Zelensky. Reference category for corrections seen variable is no corrections seen. 23
S17 Effect of corrections seen on feeling thermometer evaluations of the country Ukraine. Reference category for corrections seen variable is no corrections seen. 24
S18 Effect of corrections seen on feeling thermometer evaluations of the country Russia. Reference category for corrections seen variable is no corrections seen. 24
S19 Regression estimates for relationship between number of corrections seen and support for Russia in Ukraine war. Omitted category for corrections seen is 0 corrections seen. Dependant variable is 5pt scale, with larger values indicating support for Russia. 25
S20 Regression estimates for relationship between number of corrections seen and support for Ukraine in Ukraine war. Omitted category for corrections seen is 0 corrections seen. Dependant variable is 5pt scale, with larger values indicating support for Ukraine. 25

1 Detailed Summaries of Stimuli

Below, we present detailed summaries of the fact-checks tested in our studies. To access the stimuli in their tested form, please contact the authors.

The fact-checks are numbered for ease of exposition only; participants could have seen fact-checks in their country in any order.

Fact-checks in Belarus

Treatment 1: The first fact-check aimed at a Russian-speaking Belarusian audience refutes the misinformation that that "Ukraine is not a real state and belongs to Russia." The article that they are correcting is misinformation that was circulated on Telegram. The misinformation said that Ban Ki-moon, former General Secretary of the U.N., had claimed that Ukraine was not a real state and belonged to Russia. This line of misinformation fits within a broader slew of Russian misinformation that asserts the validity of Russian irredentism and claims over the Ukrainian state (Schwirtz, Varenikova and Gladstone, 2022; Perrigo, 2022; U.S. Department of State, 2022*b*). The fact-checking site *FactCheck.kz* makes it clear that Russian propagandists have fabricated Ban Ki-moon's statement and that Ukraine is a full member of the U.N. and that Russia has no territorial claim over Ukraine.

Treatment 2: The second fact-check aimed at a Russian-speaking Belarusian audience refutes the misinformation that the "footage of war crimes committed by Russians in Bucha, Ukraine is fake." The fact-check comes from *Deutsche Welle* (*DW*), the German News outlet. The fact-check pushes back against the widespread Russian propaganda that sought to discredit videos of war crimes in Bucha, Ukraine and claim that these videos were falsified (U.S. Department of State, 2022*a*; The German Marshall Fund, 2022). The fact check counters the narratives of pro-Russian accounts that portrayed the footage as false. *DW* clarifies that these false footage interpretations are due to optical illusions and, in fact, these were actually dead bodies in the street.

Treatment 3: The third fact-check aimed at a Russian-speaking Belarusian audience refutes the misinformation that "in Ukrainian Schools, textbooks promote Nazi ideology." The fact-check comes from *Re:Baltica*, a fact-checking agency based in Latvia. This fact-check refutes the Russian propaganda line that has been touted among Russian-backed media/ The fact-check looks at the textbooks in Kharkov, Ukraine that Russian propagandists claimed were promoting Nazi ideology. They show that the textbook focuses on Ukrainian regional security, issues of threats to Ukrainian national security, military service, and international law. The fact-check concludes that Nazism is not promoted in the textbooks, indicating that broader claims about rising Nazism in Ukraine are false (Treisman, 2022).

Fact-checks in Estonia

Treatment 1: The first fact-check aimed at a Russian-speaking Estonian audience refutes the misinformation that "Estonia is protecting Darya Dugina's murderer." This misinformation claims that Darya Dugina - the daughter of the prominent far-right political ideologue Aleksandr Dugin, whose theories have come to influence Putin's - was murdered by a Ukrainian-backed nationalist who subsequently fled to Estonia and was being harbored by the Estonian government. The fact-check, produced by Estonian fact-checker *PropaStop*, exposes the evidence as fabricated. Overall, this Russian propaganda narrative was part of a broader effort to discredit Estonia as part of a broader Ukrainian and NATO effort to target Russians (Lister,

Pavlova and Said-Moorhouse, 2022).

Treatment 2: The second fact-check aimed at a Russian-speaking Estonian audience refutes the misinformation that "Russians living in Narva, Estonia face discrimination from Estonians and need the Russian military to protect them." This claim parallels similar assertions by Russian media and leaders in Russian-dominated areas of Eastern Ukraine are in need of Russian interference to protect against Ukrainian persecution. As in Russian propaganda about Russian minorities in Eastern Ukraine, this Russian propaganda line fits with a Russian effort over many years to portray themselves as protectors of Russian minorities in the Near Abroad who need Russian assistance to counter Estonian persecution that a sizable Russian minority population in Narva, Estonia are in need of Russian help to counter Estonian persecution (McLaughlin, 2022). The Estonian fact-checking organization *PropaStop* writes that the "Russian propaganda brazenly lies" about the discrimination against Russians in Estonia as a way to paint Estonia as a threat to Russian national security and justify Russian intervention.

Treatment 3: The third fact-check aimed at a Russian-speaking Estonian audience refutes the misinformation that Estonia was responsible for sabotaging the Nord Steam pipeline in the Baltic Sea in late-September 2022. *PropaStop*, the Estonian fact-checking organization, highlights that Russian propagandists latched onto dubious social media rumors that accused Estonia of sabotage. *PropaStop* highlights how many of these social media accounts appeared to have been created around the time of the pipeline explosions, furthering the idea that Russian-backed trolls were helping to orchestrate this line of misinformation. The fact-check pushes back on one of the numerous Russian attempts to redirect blame for the explosion to Western rivals amid a time of ambiguity when the perpetrators have not yet been determined by officials and many Western officials are blaming Russia for the attack (Bodnar, 2022).

Fact-checks in Kazakhstan

Treatment 1: The first fact-check aimed at a Russian-speaking Kazakh audience refutes the misinformation by many Russian media and telegram channels that "Ukrainian refugees have burdened German schools and created a teacher shortage." The Kazakh fact-checking organization *FactCheck.kz* counters this by showing that German schools were already facing a teacher shortage prior to the influx of Ukrainian refugees. The Russian misinformation manipulates findings of research without context and selectively conveys anti-immigrant perspectives in Germany. Such a set of misinformation aligns with attempts by Russian propagandists to agitate potential areas of domestic discord around provocative issues (Morris and Oremus, 2022).

Treatment 2: The second fact-check aimed at a Russian-speaking Kazakh audience refutes the misinformation from Russian media that the separatist regions of Ukraine recognized by Russia as the Luhansk People's Republic (LNR) and the Donetsk People's Republic (DNR) have official diplomatic representation in Finland. The assertion was claimed by the pro-Russian propagandist in Finland, Johann Beckman. This claim then made its way through various Russian propaganda channels to legitimate pro-Russian separatist regions. As highlighted by the Kazakh fact-checking organization *FactCheck.kz*, no official Finnish government agency

recognizes the LNR or DNR. The only recognition comes from officials representing the LNR and DNR. The official recognition of these separatist regions comes from only a few countries and a couple partially-recognized territories - Russia, Syria, North Korea, South Ossetia, and Abkhazia. This misinformation fits within a broader slew of Russian misinformation that aims to legitimate the separatist pro-Russian regions of Ukraine and claims these regions face persecution by the Ukrainian (The U.S. Department of State, 2023).

Treatment 3: The third fact-check aimed at a Russian-speaking Kazakh audience refutes the misinformation that "Ukraine is producing fake video footage of Russian war crimes to frame Russia." This misinformation, proliferated by Russian media, state, and social media commentators, claims that Ukraine was creating of a fake film to show Russian atrocities in Ukraine. The misinformation aimed at discrediting widely known Ukrainian accusations of Russian war crimes throughout Ukraine, redirecting culpability and justifing the Russian invasion of Ukraine under the veneer of protection and "de-nazification." The misinformation circulated on Telegram channels and Russian media. But as reported by the Kazakh fact-checking organization *FactCheck.kz*, the footage was actually from a documentary production in Ukraine that was recreating specific scenes. This type of misinformation one example among numerous instances in which the Russians have sought to discredit footage of war crimes and mass death by labeling the footage as faked or orchestrated by Ukraine (Seitz and Lajka, 2022).

Fact-checks in Russia

Treatment 1: The first fact-check aimed at a Russian-speaking Russian audience refutes the misinformation that "Ukraine is trading grain exports for weapons." The misinformation mixes narratives about a modern-day Ukrainian "lend-and-lease" arrangement with the United States, in which grain is exchanged for old weapons, and other narratives about the West stealing grain to help Ukraine in the fight against Russia. In reality, Latvian fact-checking organization *Re:Baltica*, shows that this Russian misinformation aimed at supporting Russian claims of Ukrainian aggression as a Western proxy. In reality, *Re:Baltica* points out that the military aid lend-lease act is disconnected from Western aims to get Ukrainian grain out of Ukraine. Instead, because Ukraine is such a large exporter of grain, many Western leaders are calling for the need to get grain out of Ukraine in response to rising hunger and food shortages, particularly in the developing world. The Russian misinformation extends the propaganda narrative that Ukraine is a stooge of the West and receiving weapons from the West (The U.S. Department of State, 2023).

Treatment 2: The second fact-check aimed at a Russian-speaking Russian audience refutes the misinformation that "Russian troops were not involved in the deaths of civilians in Bucha." This line of misinformation directly counters the extensively-reported scenes of Russian war crimes and massacre against Ukrainians living in Bucha, Ukraine. For Russian-backed media and leaders and social media channels, this line of misinformation fits within a broader justification of Russian intervention, portraying the Ukrainians as liars, neo-Nazis, and puppets of the West. The fact-check, put forth by *Re:Baltica*, the Latvian fact-checking organization,

specifically pushes back against the misleading claim by Russian media that *The Guardian* asserted that Russia was not involved in the killings in Bucha. *Re:Baltica* demonstrates that the original article does not claim this and instead links Russian artillery to the deaths. Thus, the Russian media grossly manipulated this claim to prop up broader efforts to counter Western reports of war crimes in Bucha. This misinformation is part of a broader slew of Russian misinformation that sought to discredit the reports of Russian war crimes in Bucha and suggest that Ukrainians were falsifying the stories (Seitz and Lajka, 2022).

Treatment 2: The third fact-check aimed at a Russian-speaking Russian audience refutes the misinformation that "Russia has never started or been the aggressor in a war." This claim was asserted by Patriarch Kirill - the Russian Orthodox Church leader and Kremlin insider - and echoed by other Russian leaders. This misinformation attempts to portray Russia as a victim of foreign aggression, furthering the notion that the Western threat and the supposed Ukrainian "Nazi" government was a threat to Russian security. The Latvian fact-checking organization *Re:Baltica* refutes this claim by highlighting several historical instances in which Russia has instigated war. This effort to portray Russia as peaceful and a victim of aggression contributes to broader Russian propaganda messaging that seeks to portray Russia as a protector, justified in their war in Ukraine, provoked by Ukrainian threats and NATO expansion (Terracino and Matasick, 2022).

Fact-checks in Ukraine

Treatment 1: The first fact-check aimed at a Russian-speaking Ukrainian audience refutes the Russian misinformation that "Ukrainians deliberately murdered Russians living in the Donbas region." This misinformation is part of a broader strain of Russian propaganda that attempts to portray Ukraine as aggressors and human-rights abusers, particularly toward Russian populations living within Ukraine's boundaries, thereby, justifying the Russian invasion (Terracino and Matasick, 2022; The U.S. Department of State, 2023; Madeline Roache and Palmer, 2023). Such claims that Ukrainians commit genocide against Russian in the Donbas region have figured prominently in Russian justifications for the war. The Kazakh fact-checking organization *Factcheck.kz* pushes back on this claim, turning to evidence from OSCE observers and data on deaths in the Donbas region to counter the Russian claims, showing that no genocide has been perpetrated in the region by the Ukrainian state. Furthermore, they show that the number of deaths in the region in no way fits the legal definition of "genocide."

Treatment 2: The second fact-check aimed at a Russian-speaking Ukrainian audience refutes the misinformation that "Ukraine is raising money to buy an atomic bomb." This piece of misinformation fits in with the broader narrative about a Ukrainian threat to Russian security, Ukraine's desperation to counter the Russian offensive in the area, and nefarious Western assistance with Ukrainian weaponry (EUvsDisinfo, 2022; The Guardian, 2022). The narrative, reported on Russian-backed media and social media channels, claimed that Ukraine was trying to crowd-source money for this weapon. The fact-checking organization *Factcheck.kg* refutes this by showing that there is not a campaign to raise money for an atomic bomb.

Treatment 3: The third fact-check aimed at a Russian-speaking Ukrainian audience refutes the widespread misinformation that "the United States is creating bioweapons in Ukraine." This assertion is a widespread piece of misinformation that has been extended by countless Russian leaders, media, and social media channels since before the war in Ukraine even began (Madeline Roache and Palmer, 2023; The U.S. Department of State, 2023; Terracino and Matasick, 2022; Myers, 2022) This assertion of a U.S.-led bioweapons program in Ukraine has served as fodder for Russian propaganda to support its military offensive in Ukraine and portray Ukraine as under the influence of Western aggression and in need of Russian intervention. The fact-checking organization *Re:Baltica* counters the Russian propaganda line that the Pentagon had admitted to funding 46 biolabs in Ukraine. *Re:Baltica* shows that Russian propagandists rely on inflammatory, conspiracy ridden blog-posts to support this claim. Moreover, they highlight that there are no U.S. bio-laboratories in Ukraine and that U.S. assistance to Ukraine has been in the form of giving equipment and other standard technical help to support Ukrainian biolaboratories during the COVID-19 Pandemic.

References and Notes

Bodnar, Joseph. 2022. "A Look Into Russia's Propaganda about the Nord Stream Explosions." *The German Marshall Fund, Alliance for Securing Democracy* .
URL: *https://securingdemocracy.gmfus.org/a-look-into-russias-propaganda-about-the-nord-stream-explosions/*

EUvsDisinfo. 2022. ""ALL QUIET ON THE WESTERN FRONT"." *EUvsDisinfo* .
URL: *https://euvsdisinfo.eu/all-quiet-on-the-western-front/*

Lister, Tim, Uliana Pavlova and Lauren Said-Moorhouse. 2022. "Russian security service accuses Ukraine of Darya Dugina's murder." *CNN* .
URL: *https://www.cnn.com/2022/08/22/europe/darya-dugina-killing-russian-security-service-claim-intl/index.html*

Madeline Roache, Sophia Tewa, Alex Cadier Chine Labbe Virginia Padovese Roberta Schmid Edward O'Reilly Marie Richter Karin König McKenzie Sadeghi Chiara Vercellone Zack Fishman Natalie Adams Valerie Pavilonis Shayeza Walid Kelsey Griffin Coalter Palmer Andie Slomka Louise Vallée Akshata Kapoor Eva Maitland Macrina Wang and Kathryn Palmer. 2023. "Russia-Ukraine Disinformation Tracking Center." *News Guard* .
URL: *https://www.newsguardtech.com/special-reports/russian-disinformation-tracking-center/*

McLaughlin, Jenna. 2022. "Why the Estonian town of Narva is a target of Russian propaganda." *NPR* .
URL: *https://www.npr.org/2022/05/10/1097820850/why-the-estonian-town-of-narva-is-a-target-of-russian-propaganda*

Morris, Loveday and Will Oremus. 2022. "Russian disinformation is demonizing Ukrainian refugees." *The Washington Post* .
URL: *https://www.washingtonpost.com/technology/2022/12/08/russian-disinfo-ukrainian-refugees-germany/*

Myers, Steven Lee. 2022. "U.S. Rebukes Russia for Claims of Secret Bioweapons in Ukraine." *The New York Times* .
URL: *https://www.nytimes.com/2022/09/13/technology/russia-ukraine-bioweapons.html*

Perrigo, Billy. 2022. "How Putin's Denial of Ukraine's Statehood Rewrites History." *Time Magazine* .
URL: *https://time.com/6150046/ukraine-statehood-russia-history-putin/*

Schwirtz, Michael, Maria Varenikova and Rick Gladstone. 2022. "Putin Calls Ukrainian Statehood a Fiction. History Suggests Otherwise." *The New York Times* .
URL: *https://www.nytimes.com/2022/02/21/world/europe/putin-ukraine.html*

Seitz, Amanda and Arijeta Lajka. 2022. "Propaganda, fake videos of Ukraine invasion bombard users." *AP News* .
URL: *https://apnews.com/article/russia-ukraine-technology-europe-media-social-media-80f729025396abf9ad9e4e9d0b4f5ece*

Terracino, Julio Bacio and Craig Matasick. 2022. "Disinformation and Russia's war of aggression against Ukraine: Threats and governance responses." *Organisation for Economic Co-operation and Development* .
URL: *https://www.oecd.org/ukraine-hub/policy-responses/disinformation-and-russia-s-war-of-aggression-against-ukraine-37186bde/*

The German Marshall Fund. 2022. "Russia pushes disinformation about the Bucha massacre." *The German Marshall Fund, Alliance for Securing Democracy* .
URL: *https://securingdemocracy.gmfus.org/incident/russia-pushes-disinformation-about-the-bucha-massacre/*

The Guardian. 2022. "US dismisses 'transparently false' Russian claims of Ukraine plan to use 'dirty bomb'." *The Guardian* .
URL: *https://www.theguardian.com/world/2022/oct/24/us-dismisses-transparently-false-russian-claims-of-ukraine-plan-to-use-dirty-bomb*

The U.S. Department of State. 2023. "Disinformation Roulette: The Kremlin's Year of Lies to Justify an Unjustifiable War." *Global Engagement Center, the U.S. Department of State* .
URL: *https://www.state.gov/disarming-disinformation/disinformation-roulette-the-kremlins-year-of-lies-to-justify-an-unjustifiable-war/*

Treisman, Rachel. 2022. "Putin's claim of fighting against Ukraine 'neo-Nazis' distorts history, scholars say." *NPR* .
URL: *https://www.npr.org/2022/03/01/1083677765/putin-denazify-ukraine-russia-history*

U.S. Department of State. 2022*a*. "Russian Federation Disinformation About Its Atrocities in Bucha.".
URL: *https://www.state.gov/disarming-disinformation/russian-federation-disinformation-about-its-atrocities-in-bucha/*

U.S. Department of State. 2022*b*. "Russia's War on Ukraine: Six Months of Lies, Implemented.".
URL: *https://www.state.gov/disarming-disinformation/russias-war-on-ukraine-six-months-of-lies-implemented/*

2 Tables

Table S1: Popularity of the Fact-Checking Websites

Misinformation	Fact-Checking Website	Popularity
Ukraine is not a real state, belongs to Russia	factcheck.kz	3.8K
Footage of Russian war crimes in Bucha is faked	Deutsche Welle	8.3M
In Ukrainian schools, textbooks promote Nazism	Re:Baltica	4.6K
Estonia is protecting Darya Dugina's murderer	PropaStop	3.3K
Russians living in Narva, Estonia, face discrimination	PropaStop	3.3K
Estonia sabotaged the Nord Stream pipeline	PropaStop	3.3K
Ukrainian refugees have created a teacher shortage	factcheck.kz	3.8K
Finland recognizes Luhansk and Donetsk Republics	factcheck.kz	3.8K
Ukraine produces fake videos of Russian war crimes	factcheck.kz	3.8K
Ukraine trades in grain in exchange for weapons	Re:Baltica	4.6K
Russian troops not involved in Bucha civilian deaths	Re:Baltica	4.6K
Russia has never started or been the aggressor in a war	Re:Baltica	4.6K
Ukrainians deliberately murdered Russian civilians in Donbas	factcheck.kg	2.9K
Ukraine raising money for an atomic bomb	factcheck.kg	2.9K
US creates bio-weapons in Ukraine	Re:Baltica	4.6K

Popularity data is measured by organic traffic, which is the estimated number of monthly visits a target website receives from organic search results. Data for this analysis was obtained from Ahrefs' Traffic Checker tool (https://ahrefs.com/traffic-checker).

Table S2: Prevalence of Misinformation

Misinformation	Underlying Misinformation	Data about Underlying Misinformation Popularity
Ukraine is not a real state, belongs to Russia	https://vk.com/wall-96930993_575604	12K views of the post on Vkontakte
Footage of Russian war crimes in Bucha is faked	https://x.com/mfa_russia/status/1512159243134242821?s=20	5.2K engagements with the post on Twitter
In Ukrainian schools, textbooks promote Nazism	https://t.me/tass_agency/130789	60.3K views of the post on Telegram
Estonia is protecting Darya Dugina's murderer	https://t.me/tsargradtv/22267	24.8K views of the post on Telegram
Russians living in Narva, Estonia, face discrimination	https://www.pravda.ru/world/1733207-narva_russia/	4.4K views of the post on Telegram
Estonia sabotaged the Nord Stream pipeline	https://twitter.com/BBCr4today/status/1514549851035516934?s=20&t=vgMC8_Z_jnbqEvKByXstfA	17 engagements with the post on Twitter
Ukrainian refugees have created a teacher shortage	https://t.me/swodki/146710	44.3K views of the post on Telegram
Finland recognizes Luhansk and Donetsk Republics	https://www.1tv.ru/shows/vremya-pokazhet/vypuski/vremya-pokazhet-chast-1-vypusk-ot-29-06-2022	5,865 followers of Johan Backman, who presents himself as the official representative of the LPR and DPR in Finland, on Twitter
Ukraine produces fake videos of Russian war crimes	https://t.me/SolovievLive/120877	218.1K views of the post on Telegram
Ukraine trades in grain in exchange for weapons	https://t.me/vestiru24/32825	29.5K views of the post on Telegram
Russian troops not involved in Bucha civilian deaths	https://t.me/readovkaru/364	347.4K views of the post on Telegram
Russia has never started or been the aggressor in a war	https://ria.ru/20220503/patriarkh-1786640294.html	6,917 views of the news on RIA
Ukrainians deliberately murdered Russian civilians in Donbas	https://ria.ru/20211209/putin-1763032349.html	93,728 views of the news on RIA
Ukraine raising money for an atomic bomb	https://t.me/rlz_the_kraken/50854	94.6K views of the post on Telegram
US creates bio-weapons in Ukraine	https://www.youtube.com/watch?app=desktop&v=VnfS4sBbjl4	168K views of the video on YouTube

	Corrections Seen			
	0	1	2	3
Ukraine	266	250	247	254
Russia	228	227	241	249
Kazakhstan	234	222	204	223
Belarus	185	180	183	179
Estonia	102	99	79	94

Table S3: The number of survey participants by country and number of corrections assigned.

Table S4: Summary Age Demographic in Survey Compared with Population Estimates

Country	Survey (19-65)	Survey %	Pop. Est. (20-64)	Pop. %	Survey (66+)	Survey %	Pop. Est (65+)	Pop. %
BLR	702	99.01%	5 879 000	78.48%	7	0.99%	1 612 000	21.52%
EST	354	95.93%	773 000	74.04%	15	4.07%	271 000	25.96%
KAZ	832	99.28%	10 702 000	87.52%	6	0.72%	1 526 000	12.48%
RUS	900	96.36%	89 422 000	79.81%	34	3.64%	22 626 000	20.19%
UKR	969	98.88%	27 288 000	78.27%	11	1.12%	7 574 000	21.73%

Population data come from 2021 United Nations estimates. See: United Nations, Department of Economic and Social Affairs, Population Division (2022). World Population Prospects 2022, Online Edition, https://population.un.org/wpp/Download/Standard/Population/.

Table S5: Summary Gender Demographic in Survey Compared with Population Estimates

Country	% Female, Population	% Female, Sample	Diff (%)
BLR	54.0%	36.0%	-18.0%
EST	52.5%	50.0%	-2.5%
KAZ	51.9%	38.0%	-13.9%
RUS	53.6%	34.0%	-19.6%
UKR	54.1%	49.0%	-5.1%

Population data come from 2021 United Nations estimates. See: United Nations, Department of Economic and Social Affairs, Population Division (2022). World Population Prospects 2022, Online Edition, https://population.un.org/wpp/Download/Standard/Population/.

Table S6: Summary comparing survey regional demographics with real Belarusian regional demographics

Region	Survey Number	Survey Percent	Pop. Est.	Pop. Est. Percent
Brest Region	87	12.24%	1,379,456	14.59%
Gomel Region	106	14.91%	1405873	14.87%
Grodno Region	52	7.31%	1036529	10.96%
Minsk Region	284	39.94%	3419052	36.16%
Mogilev Region	69	9.70%	1049326	11.10%
Vitebsk Region	102	14.35%	1,164,554	12.32%
I live in a different place	11	1.55%	N/A	N/A

Data Belarusian census in 2019. See: https://www.belstat.gov.by

Table S7: Summary comparing survey regional demographics with real Estonian regional demographics

Region	Survey Number	Survey Percent	Pop. Est.	Pop. Est. Percent
Harju County	179	48.38%	614,561	46.15%
Hiiu County	5	1.35%	8,497	0.64%
Ida-Viru County	49	13.24%	132,736	9.97%
Järva County	5	1.35%	27,857	2.09%
Jõgeva County	7	1.89%	29,693	2.23%
Lääne County	7	1.89%	20,227	1.52%
Lääne-Viru County	10	2.70%	58,709	4.41%
Pärnu County	18	4.86%	23,989	1.80%
Põlva County	3	0.81%	85,705	6.44%
Rapla County	6	1.62%	33,529	2.52%
Saare County	6	1.62%	31,292	2.35%
Tartu County	46	12.43%	157,758	11.85%
Valga County	5	1.35%	27,650	2.08%
Viljandi County	9	2.43%	45,411	3.41%
Võru County	8	2.16%	34,182	2.57%
I live in a different place	7	1.89%	N/A	N/A

Data Estonian 2021 government census. See:
https://www.stat.ee/en/find-statistics/statistics-by-region

Table S8: Summary comparing survey regional demographics with real Kazakh regional demographics

Region	Survey Number	Survey Percent	Pop. Est.	Pop. Est. Percent
Abai Region	8	0.95%	610,010	3.08%
Akmola Region	52	6.20%	788,119	3.98%
Aktobe Region	24	2.86%	929,245	4.70%
Almaty (city)	202	24.08%	2,167,363	10.95%
Almaty Region	42	5.01%	1,508,169	7.62%
Astana (city)	104	12.40%	1,359,583	6.87%
Atyrau Region	13	1.55%	694,132	3.51%
East Kazakhstan Region	53	6.32%	730,037	3.69%
Jambyl Region	19	2.26%	1218609	6.16%
Jetisu Region	3	0.36%	698,569	3.53%
Karaganda Region	53	6.32%	1,134,873	5.74%
Kostanay Region	54	6.44%	831,854	4.20%
Kyzylorda Region	11	1.31%	834,552	4.22%
Mangystau Region	11	1.31%	768,930	3.89%
North Kazakhstan Region	46	5.48%	533,627	2.70%
Pavlodar Region	57	6.79%	754,890	3.81%
Shymkent (city)	34	4.05%	1,194,106	6.03%
Turkistan Region	11	1.31%	2,121,672	10.72%
Ulytau Region	5	0.60%	221,401	1.12%
West Kazakhstan Region	31	3.69%	688,513	3.48%
I live in a different place	6	0.72%	N/A	N/A

Data from 2023 Kazakh government regional data. See: https://new.stat.gov.kz/ru/region/

Table S9: Survey regional demographics vs. Russian regional demographics (data from 2021 Russian census. See: https://rosstat.gov.ru)

Region	Survey Number	Survey Percent	Pop. Est.	Pop. Est. Percent
Amur Region	6	0.64%	766,912	0.53%
Arkhangelsk Region	3	0.32%	978,873	0.68%
Astrakhan Region	4	0.43%	960,142	0.66%
Belgorod Region	10	1.07%	1,540,486	1.06%
Bryansk Region	6	0.64%	1,169,161	0.81%
Chelyabinsk Region	21	2.24%	3,431,224	2.37%
Irkutsk Region	17	1.82%	2,370,102	1.64%
Ivanovo Region	11	1.18%	927,828	0.64%
Kaliningrad Region	2	0.21%	1,029,966	0.71%
Kaluga Region	7	0.75%	1,069,904	0.74%
Kemerovo Region	19	2.03%	2,600,923	1.80%
Kirov Region	7	0.75%	1,153,680	0.80%
Kostroma Region	3	0.32%	580,976	0.40%
Kurgan Region	2	0.21%	776,661	0.54%
Kursk Region	5	0.53%	1,082,458	0.75%
Leningrad Region	68	7.26%	2,000,997	1.38%
Lipetsk Region	4	0.43%	1,143,224	0.79%
Magadan Region	1	0.11%	136,085	0.09%
Moscow Region	216	23.08%	8,524,665	5.89%
Murmansk Region	5	0.53%	667,744	0.46%
Nizhny Novgorod Region	28	2.99%	3,119,115	2.16%
Novgorod Region	3	0.32%	583,387	0.40%
Novosibirsk Region	30	3.21%	2,797,176	1.93%
Omsk Region	12	1.28%	1,858,798	1.28%
Orenburg Region	10	1.07%	1,862,767	1.29%
Oryol Region	5	0.53%	713,374	0.49%
Penza Region	7	0.75%	1,266,348	0.88%
Pskov Region	5	0.53%	599,084	0.41%
Rostov Region	35	3.74%	4,200,729	2.90%
Ryazan Region	7	0.75%	1,102,810	0.76%
Sakhalin Region	3	0.32%	466,609	0.32%
Samara Region	38	4.06%	3,172,925	2.19%
Saratov Region	21	2.24%	2,442,575	1.69%
Smolensk Region	6	0.64%	888,421	0.61%
Sverdlovsk Region	49	5.24%	4,268,998	2.95%
Tambov Region	7	0.75%	982,991	0.68%
Tomsk Region	6	0.64%	1,062,666	0.73%
Tula Region	11	1.18%	1,501,214	1.04%
Tver Region	4	0.43%	1,230,171	0.85%
Tyumen Region	14	1.50%	3,823,910	2.64%
Ulyanovsk Region	9	0.96%	1,196,745	0.83%
Vladimir Region	6	0.64%	1,348,134	0.93%
Volgograd Region	19	2.03%	2,500,781	1.73%
Vologda Region	10	1.07%	1,142,827	0.79%
Voronezh Region	20	2.14%	2,308,792	1.60%
Yaroslavl Region	6	0.64%	1,209,811	0.84%
I live in a different place	148	15.81%	N/A	N/A

Table S10: Survey regional demographics vs. Ukrainian regional demographics

Region	Survey Number	Survey Percent	Pop. Est.	Pop. Est. Percent
Autonomous Republic of Crimea	1	0.10%	1,916,805	4.45%
Cherkasy Region	31	3.14%	1,159,200	2.69%
Chernihiv Region	28	2.84%	957,665	2.22%
Chernivtsi Region	19	1.93%	889,928	2.07%
Dnipropetrovsk Region	128	12.98%	3,093,151	7.19%
Donetsk Region	2	0.20%	4,056,405	9.42%
Ivano-Frankivsk Region	23	2.33%	1,350,565	3.14%
Kharkiv Region	87	8.82%	2,596,250	6.03%
Kherson Region	0	0.00%	1,000,370	2.32%
Khmelnytskyi Region	30	3.04%	1,227,474	2.85%
Kiev (city)	164	16.63%	2,950,702	6.85%
Kiev Region	47	4.77%	1,795,542	4.17%
Kirovohrad Region	26	2.64%	902,275	2.10%
Luhansk Region	0	0.00%	2,101,653	4.88%
Lviv Region	67	6.80%	2,476,113	5.75%
Mykolaiv Region	23	2.33%	1,090,492	2.53%
Odessa Region	64	6.49%	2,349,749	5.46%
Poltava Region	45	4.56%	1,350,564	3.14%
Rivne Region	20	2.03%	1,140,902	2.65%
Sumy Region	31	3.14%	1,034,364	2.40%
Ternopil Region	19	1.93%	1,020,953	2.37%
Vinnytsia Region	37	3.75%	1,507,738	3.50%
Volyn Region	16	1.62%	1,020,770	2.37%
Zakarpattia Region	9	0.91%	1,243,721	2.89%
Zaporizhia Region	38	3.85%	1,636,322	3.80%
Zhytomyr Region	24	2.43%	1,177,564	2.74%
I live in a different place	7	0.71%	N/A	N/A

Data are estimates of Ukrainian population in 2022 from Statista (most recent official census was in 2001 and has been postponed until 2023 due to Russian invasion). See: https://www.statista.com/statistics/1295222/ukraine-population-by-region/

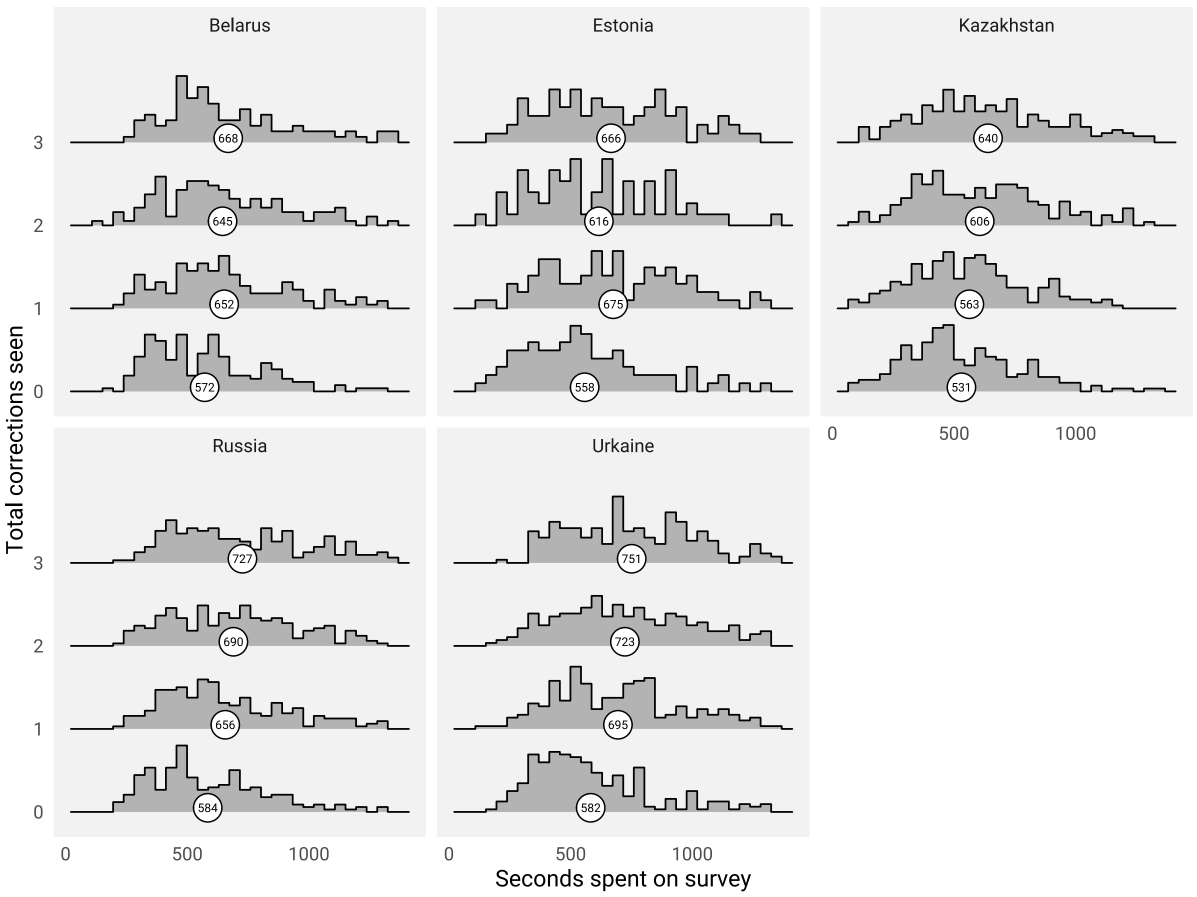

Figure S1: Time spent by participants inside the survey environment, by country and corrections seen. Labeled points report the mean duration spent on the survey.

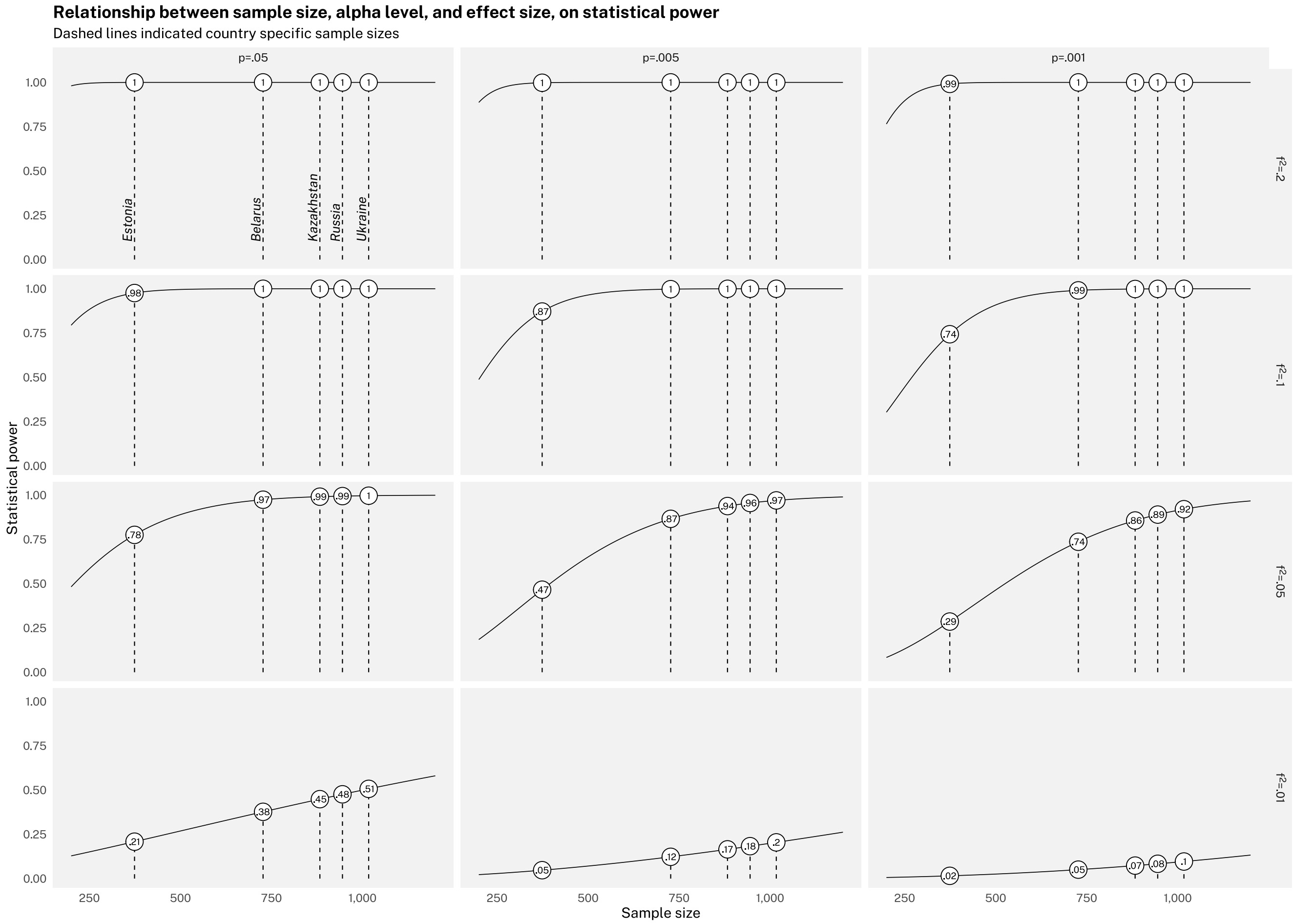

Figure S2: The statistical power for three levels of significance (indicated in column facets) and four levels of effect size (indicated by the row facets). The statistical power observed in each country is indicated by the labeled points for every combination of significance level and effect size.

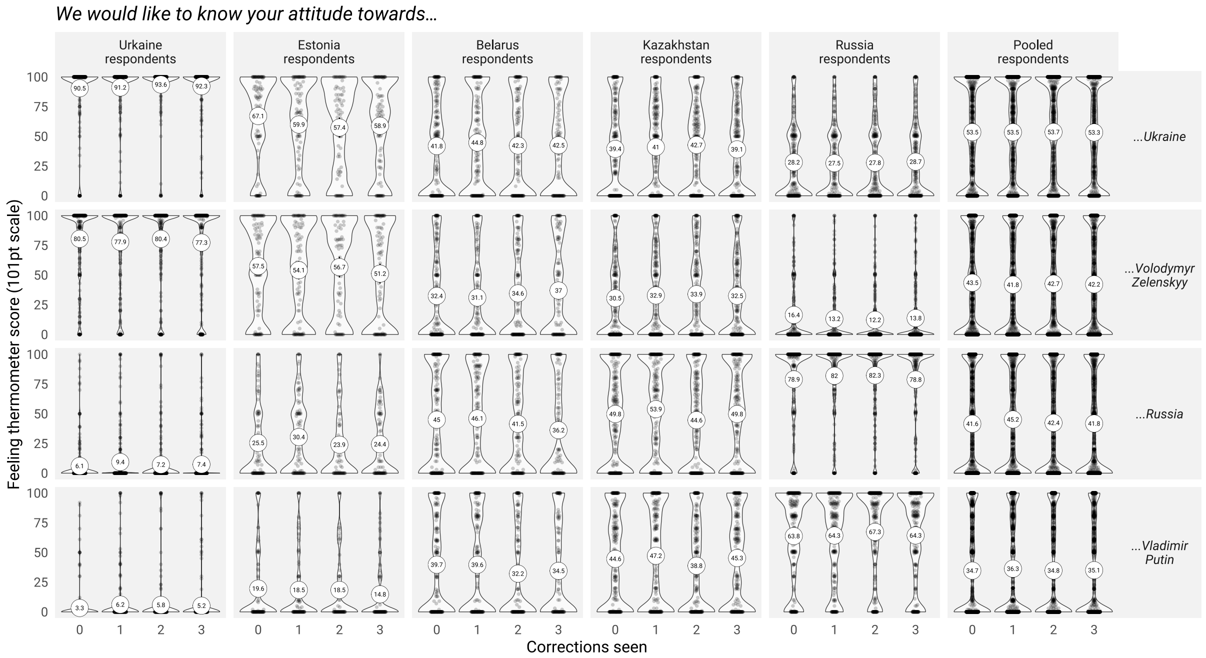

Figure S3: Feeling thermometer evaluations by country. Labels indicate mean evaluations.

	Belarus			Estonia			Kazakhstan			Russia			Ukraine		
	FC 1	FC 2	FC 3	FC 1	FC 2	FC 3	FC 1	FC 3	FC 2	FC 1	FC 2	FC 3	FC 2	FC 1	FC 3
Treatment	-0.03	-0.44***	-0.2	0.01	-0.05	0.04	-0.06	0.24**	-0.05	-0.52***	-0.35***	-0.06	-0.91***	-0.77***	0.02
	(0.1)	(0.11)	(0.12)	(0.12)	(0.1)	(0.12)	(0.08)	(0.09)	(0.08)	(0.09)	(0.08)	(0.09)	(0.05)	(0.05)	(0.060
Num.Obs.	723	724	581	373	372	371	882	878	880	943	943	942	992	998	1010
R2	0.0001	0.022	0.005	0.0001	0.001	0.0001	0.001	0.008	0.0001	0.032	0.02	0.0001	0.285	0.182	0.0001

Note: * p <.05, ** p <.01, *** p <.001

Table S11: Regression estimates for correction effects. Treatment effects report the difference in factual accuracy of seeing a factual correction. The dependant quality is measured on a 5-pt scale, with higher values indicating less accurate beliefs. Intercepts are omitted from this table for simplicity, These models underpin the correction estimates in Figure 3.

Table S12: Meta analytic summaries of agreement with misinformation among subjects in control condition

	Overall	Belarus	Estonia	Kazakhstan	Russia	Ukraine
Overall	2.63***	2.64***	1.86***	2.86***	3.91***	1.87***
	(0.22)	(0.31)	(0.16)	(0.23)	(0.06)	(0.24)
Num.Obs.	15	3	3	3	3	3
AIC	39.11	7.23	4.44	6.02	0.84	6.2
BIC	40.38	4.62	1.82	3.4	-1.77	3.59
Log.Lik.	-17.553	-1.616	-0.218	-1.009	1.578	-1.102

Note: * $p < .05$, ** $p < .01$, *** $p < .001$

Table S13: Meta analytic summaries of agreement with misinformation among subjects in correction condition

	Overall	Belarus	Estonia	Kazakhstan	Russia	Ukraine
Treatment	2.42***	2.42***	1.86***	2.9***	3.61***	1.31***
	(0.23)	(0.20)	(0.17)	(0.31)	(0.19)	(0.06)
Num.Obs.	15	3	3	3	3	3
AIC	40.04	5.36	4.82	7.17	5.21	0.54
BIC	41.31	2.75	2.21	4.56	2.6	-2.07
Log.Lik.	-18.018	-0.681	-0.41	-1.587	-0.605	1.728

Note: * $p < .05$, ** $p < .01$, *** $p < .001$

Table S14: Meta analytic summaries of difference in agreement between conditions, overall, and by country

	Overall	Belarus	Estonia	Kazakhstan	Russia	Ukraine
Treatment	-0.212*	-0.222	-0.003	0.042	-0.307*	-0.552
	(0.086)	(0.122)	(0.066)	(0.096)	(0.133)	(0.289)
Num.Obs.	15	3	3	3	3	3
Log.Lik.	-4.295	0.287	2.317	0.724	0.082	-1.453

* $p < 0.05$, ** $p < 0.01$, *** $p < 0.001$

Table S15: Effect of corrections seen on feeling thermometer evaluations of Vladimir Putin. Reference category for corrections seen variable is no corrections seen.

	Pooled	Ukraine	Russia	Belarus	Kazakhstan	Estonia
Intercept	34.7***	3.28***	63.8***	39.71***	44.61***	19.62***
	(1.27)	(0.84)	(2.51)	(3.03)	(2.58)	(3.21)
Corrections seen = 1	1.63	2.88	0.45	-0.08	2.63	-1.16
	(1.84)	(1.51)	(3.48)	(4.49)	(3.77)	(4.43)
Corrections seen = 2	0.11	2.56	3.53	-7.53	-5.79	-1.17
	(1.87)	(1.58)	(3.47)	(4.56)	(3.73)	(4.69)
Corrections seen = 3	0.37	1.94	0.53	-5.2	0.69	-4.83
	(1.84)	(1.44)	(3.45)	(4.91)	(3.68)	(4.23)
Num.Obs.	3764	1005	938	578	875	368
R2	0.000	0.004	0.001	0.007	0.006	0.004

* $p < 0.05$, ** $p < 0.01$, *** $p < 0.001$

Table S16: Effect of corrections seen on feeling thermometer evaluations of Volodymyr Zelensky. Reference category for corrections seen variable is no corrections seen.

	Pooled	Ukraine	Russia	Belarus	Kazakhstan	Estonia
Intercept	43.52***	80.51***	16.43***	32.43***	30.53***	57.53***
	(1.3)	(1.91)	(1.83)	(2.75)	(2.22)	(3.81)
Corrections seen = 1	-1.67	-2.6	-3.27	-1.36	2.32	-3.41
	(1.86)	(2.89)	(2.42)	(4.02)	(3.25)	(5.51)
Corrections seen = 2	-0.79	-0.09	-4.19	2.15	3.38	-0.83
	(1.9)	(2.85)	(2.36)	(4.27)	(3.31)	(5.86)
Corrections seen = 3	-1.33	-3.18	-2.68	4.57	1.98	-6.32
	(1.89)	(2.96)	(2.42)	(4.65)	(3.22)	(5.7)
Num.Obs.	3764	1005	938	578	875	368
R2	0.000	0.002	0.004	0.003	0.001	0.004

* $p < 0.05$, ** $p < 0.01$, *** $p < 0.001$

Table S17: Effect of corrections seen on feeling thermometer evaluations of the country Ukraine. Reference category for corrections seen variable is no corrections seen.

	Pooled	Ukraine	Russia	Belarus	Kazakhstan	Estonia
Intercept	53.51***	90.48***	28.21***	41.83***	39.43***	67.1***
	(1.3)	(1.58)	(2.04)	(2.9)	(2.47)	(3.56)
Corrections seen = 1	-0.05	0.68	-0.75	2.93	1.53	-7.15
	(1.85)	(2.18)	(2.79)	(4.23)	(3.48)	(5.2)
Corrections seen = 2	0.15	3.15	-0.42	0.43	3.27	-9.67
	(1.9)	(2.06)	(2.86)	(4.53)	(3.57)	(5.57)
Corrections seen = 3	-0.22	1.77	0.46	0.68	-0.37	-8.19
	(1.88)	(2.13)	(2.84)	(4.83)	(3.45)	(5.3)
Num.Obs.	3764	1005	938	578	875	368
R2	0.000	0.003	0.000	0.001	0.001	0.010

* $p < 0.05$, ** $p < 0.01$, *** $p < 0.001$

Table S18: Effect of corrections seen on feeling thermometer evaluations of the country Russia. Reference category for corrections seen variable is no corrections seen.

	Pooled	Ukraine	Russia	Belarus	Kazakhstan	Estonia
Intercept	41.57***	6.07***	78.94***	44.98***	49.77***	25.47***
	(1.33)	(1.06)	(2.19)	(3.04)	(2.6)	(3.13)
Corrections seen = 1	3.64	3.3	3.04	1.09	4.13	4.95
	(1.91)	(1.77)	(2.93)	(4.44)	(3.76)	(4.46)
Corrections seen = 2	0.78	1.1	3.37	-3.5	-5.17	-1.52
	(1.95)	(1.71)	(2.9)	(4.58)	(3.81)	(4.72)
Corrections seen = 3	0.21	1.31	-0.19	-8.79	-0.01	-1.09
	(1.92)	(1.6)	(3.04)	(4.95)	(3.73)	(4.23)
Num.Obs.	3764	1005	938	578	875	368
R2	0.001	0.004	0.003	0.008	0.007	0.007

* $p < 0.05$, ** $p < 0.01$, *** $p < 0.001$

Table S19: Regression estimates for relationship between number of corrections seen and support for Russia in Ukraine war. Omitted category for corrections seen is 0 corrections seen. Dependant variable is 5pt scale, with larger values indicating support for Russia.

	Pooled	Ukraine	Russia	Belarus	Kazakhstan	Estonia
Intercept	2.19***	1.19***	3.72***	2.19***	2.18***	1.36***
	(0.06)	(0.05)	(0.12)	(0.14)	(0.12)	(0.11)
Corrections seen = 1	-0.01	0.06	-0.1	-0.07	-0.06	0.06
	(0.08)	(0.08)	(0.16)	(0.2)	(0.17)	(0.16)
Corrections seen = 2	0.03	0.03	0.15	-0.29	-0.23	0.07
	(0.08)	(0.08)	(0.16)	(0.21)	(0.17)	(0.18)
Corrections seen = 3	-0.05	-0.01	-0.14	-0.43*	-0.09	0.04
	(0.08)	(0.08)	(0.16)	(0.21)	(0.17)	(0.16)
Num.Obs.	3764	1005	938	578	875	368
R2	0.000	0.001	0.004	0.008	0.002	0.001

* $p < 0.05$, ** $p < 0.01$, *** $p < 0.001$

Table S20: Regression estimates for relationship between number of corrections seen and support for Ukraine in Ukraine war. Omitted category for corrections seen is 0 corrections seen. Dependant variable is 5pt scale, with larger values indicating support for Ukraine.

	Pooled	Ukraine	Russia	Belarus	Kazakhstan	Estonia
Intercept	2.78***	4.5***	1.7***	2.19***	2.02***	3.48***
	(0.06)	(0.08)	(0.09)	(0.14)	(0.12)	(0.18)
Corrections seen = 1	-0.04	-0.12	-0.18	-0.03	0.25	-0.14
	(0.09)	(0.12)	(0.12)	(0.2)	(0.17)	(0.26)
Corrections seen = 2	-0.08	-0.05	-0.08	0.16	-0.12	-0.25
	(0.09)	(0.12)	(0.13)	(0.22)	(0.17)	(0.28)
Corrections seen = 3	0.05	0.06	-0.12	0.21	0.06	0.19
	(0.09)	(0.11)	(0.12)	(0.23)	(0.17)	(0.26)
Num.Obs.	3764	1005	938	578	875	368
R2	0.001	0.003	0.002	0.003	0.005	0.007

* $p < 0.05$, ** $p < 0.01$, *** $p < 0.001$

3 Pre-registration

ASPREDICTED

Wharton CREDIBILITY LAB
UNIVERSITY of PENNSYLVANIA

CONFIDENTIAL - FOR PEER-REVIEW ONLY

Factual Corrections in the Former Soviet Union (#112989)

Created: 11/14/2022 01:49 PM (PT)

This is an anonymized copy (without author names) of the pre-registration. It was created by the author(s) to use during peer-review.
A non-anonymized version (containing author names) should be made available by the authors when the work it supports is made public.

1) Have any data been collected for this study already?
No, no data have been collected for this study yet.

2) What's the main question being asked or hypothesis being tested in this study?
H1: Factual corrections will increase belief accuracy.

3) Describe the key dependent variable(s) specifying how they will be measured.
The factual belief questions will come during each corresponding trial (e.g., when a trial in Belarus involves a fact-check concerning false claims that Ukraine belongs to Russia, the factual belief statement "Ukraine is not a real state and belongs to Russia" will be presented during that trial). All attitudinal questions, including feeling thermometers, will come after all three trials have been concluded.

Factual Belief DVs: Our first set of DV's are about the factual beliefs that people hold. In our 5-country panel study, we test a total of 15 factual beliefs. We hypothesize that exposure to the treatment condition will affect people's factual beliefs. We measure this using a 5 point scale (1=completely agree, 5= disagree). ⍰
Attitudinal DVs: In addition to factual beliefs, other DV's include attitudes toward Russia and Ukraine. We will see if exposure to the treatment influences (or doesn't influence) support for the war in Ukraine and which side they support. This is measured on a 1-5 point scale, where 1=strongly agree and 5=disagree. Attitude questions will be asked at the end of all three trials.

Finally, our attitudinal DVs include feeling thermometers to various subjects related to the current war in Ukraine. We will see if exposure to the factual corrections alters feelings toward these entities. This feeling thermometer is measured on a scale of 0-100. 0=cold or negative feelings, 50=neutral, and 100=hot or positive feelings.

4) How many and which conditions will participants be assigned to?
In each country, participants will be enrolled in three trials. In each trial, participants will be randomly assigned to either a factual correction or control. Trials are order-randomized.

5) Specify exactly which analyses you will conduct to examine the main question/hypothesis.
To evaluate H1, we will provide a meta-analytic summary of separate OLS regressions with cluster-robust standard errors for each experimental trial within each country, with the factual belief questions serving as the dependent variables.

To evaluate RQ1a, we will re-scale the two attitudinal items about support so that higher numbers indicate support for Ukraine. We will then provide a meta-analytic summary of separate OLS regressions with cluster-robust standard errors for each country, with the attitude index serving as the dependent variable. The independent variable will be a count variable of the number of corrections seen (0-3).

To evaluate RQ1b, we average responses toward Ukraine and Zelensky, and, separately, to Russia and Putin. We will then provide a meta-analytic summary of separate OLS regressions with cluster-robust standard errors for each country, with the averages serving as the dependent variable. The independent variable will be a count variable of the number of corrections seen (0-3).

To evaluate RQ2, we will compare the meta-analytic estimates of the factual belief outcomes for each country, considering every dyad except those involving Estonia, as we do not expect to have sufficient statistical power there. We will also compare the attitudinal outcomes between every country, and we will compare the Russian/Putin and Ukraine/Zelensky feeling thermometer responses for each country. We will then use the Benjamini Yekutieli (2001) procedure to adjust for multiple comparisons.

Regression estimates will be provided by the lm_robust function in the estimatr R library. Meta analytic summaries will be provided by the rma.uni function in the metafor R library.   Alpha will be .05. All analyses will be two-tailed.

6) Describe exactly how outliers will be defined and handled, and your precise rule(s) for excluding observations.
Participants who fail a pre-treatment attention check will be prevented from participating further in the study.

7) How many observations will be collected or what will determine sample size? No need to justify decision, but be precise about exactly how the

number will be determined.
Russia – 1000 completes
Ukraine – 1000 completes
Belarus – 1000 completes
Estonia – 200-300 completes
Kazakhstan – 1000 completes

These are the maximum sample sizes that are achievable by our vendor.

8) Anything else you would like to pre-register? (e.g., secondary analyses, variables collected for exploratory purposes, unusual analyses planned?)
RQ1a: Will attitudes toward the War in Ukraine, its legitimacy, and support for Russia or Ukraine in the war change due to the number of factual corrections seen?

RQ1b: Will people's feeling thermometer views toward Russia and Ukraine, and their leaders, change due to the number of factual corrections seen?

RQ2: How do these effects differ (or not differ) between the countries in our panel?

We are also interested in investigating how language spoken in respondents' homes affects outcome questions. If approximately half the sample reports speaking another language at home, besides Russian, we will investigate heterogenous effects by language. To do so, we will create indicator variables for Russian language spoken exclusively at home, and interact that variable with treatment assignment, to provide meta-analytic estimates for our factual belief outcomes and our attitude questions.
